# Time to revisit Dr Finlay's casebook? The unique potential of the general practice case report

**DOI:** 10.1186/1757-1626-2-119

**Published:** 2009-02-03

**Authors:** Christian D Mallen

**Affiliations:** 1 Arthritis Research Campaign National Primary Care Centre, Keele University, Keele, Staffordshire, ST5 5BG, UK

## 

Case reports have played a crucial role throughout the history of medicine. They have been used to describe rare and exceptional diseases, illustrate diagnostic or therapeutic procedures, challenges, actions, errors, and their outcomes [1,2]. In the modern era of evidence-based medicine, large-scale population based research and randomised clinical trials we ought not to mourn the passing of eponymous diseases and authoritarian diatribes. But there is still something deeply engaging in the case report that provides a detailed account of an individual patient. It is a form of sharing medical knowledge that general practitioners ought to be at the forefront of. Furthermore it is within the grasp of each and every general practitioner to make their contribution. So what's holding us back?

Cases seen in general practice are frequently seen as being routine and unspectacular. Whilst this may be true in terms of their actual diagnosis, when other components, such as multimorbidity, social disadvantage, psychological characteristics, are added into the equation the unique potential of general practice cases is realised. More than any other group general practitioners are able to appreciate illness (the ill health a patient identifies themselves with, usually based on self-reported psychological or physical symptoms) rather than disease (the biological process that doctors use to explain and understand illness) [3], with knowledge of the patient over time and of his or her family and community. This information represents an essential component to the successful management of our patients, yet these components could never be captured in a randomised controlled trial or large prospective cohort.

It is not as if general practitioners are unaware of the importance and usefulness of case reports. They have been used as teaching instruments for many years and the Royal College of General Practitioners have recently introduced case-based discussions as a core part of the Workplace Based Assessment (WPBA) component of the new Membership of the Royal College of General Practitioners (nMRCGP) examination [4]. Yet in primary care we fail to have a strong tradition of publishing our interesting cases, unlike our secondary care colleagues. In a recent review of the first 100 cases published in the Journal of Case Reports not one was contributed by a GP despite the editor being a general practitioner and a Professor of Family Medicine [5]. This is disappointing as most patients' journey starts (and ends) in primary care, often with undifferentiated symptoms that take time to reveal themselves. This filtering and interpretation of multiple physical symptoms, entwined with social and psychological complexities, forms a significant part of clinical practice and only the generalist can fully appreciate the challenges associated with successful management of such patients.

Until now one barrier to publishing cases in primary care has been the lack of a suitable outlet. Specialist journals thrive on the 'weird and the wonderful' case reports submitted to them by secondary care clinicians. General practice has had fewer options for publishing case reports and so we should welcome a new open access journal with a philosophy that encourages us to share our wealth of experience. Observation of individuals in clinical practice has formed the basis of clinical epidemiology and general practitioners, who see around 30 patients a day, collect a myriad of such observations every week. Other editorials published by this journal have noted that for a 'case bank' to be truly successful, and informative to clinical care, cases need to be available in huge numbers [5-7]. This does not seem possible without significant general practice input.

As general practitioners, we share cases with our clinical partners everyday and we all learn from these informal experiences. Despite the potential of sharing this with a wider audience, GPs have no real tradition of publishing case reports. Whilst our colleagues in secondary care often publish for career advancement, primary care physicians do not. Time in general practice is an important commodity, and being self employed often results in a 'time is money' scenario in which publishing case reports may be considered to be a low priority. Fortunately new incentives for general practitioners to publish case reports are emerging. The creation of dedicated Academic Vocational Training Schemes [8] will produce a new generation of research-aware clinicians and the introduction of revalidation and a self-nomination process for Fellowship of the Royal College of General Practitioners may encourage established clinicians to submit their case reports for publication, a process that is facilitated by the use of a standardised proforma and the innovative use of video case reports by Cases Journal.

In the 1960's, one of the most popular television programmes broadcast on British television was Dr Finlay's casebook, written by Dr AJ Cronin [9]. Every week millions of people tuned into watch the daily medical needs of the sleepy lowland community of Tannochbrae in rural Scotland [9]. If Dr Finlay was willing to share his casebook with you, shouldn't we all be returning the favour?
